# Association of Gut Microbiota-Related Metabolites and Type 2 Diabetes in Two Puerto Rican Cohorts

**DOI:** 10.3390/nu16070959

**Published:** 2024-03-27

**Authors:** Caleigh M. Sawicki, Lorena S. Pacheco, Sona Rivas-Tumanyan, Zheyi Cao, Danielle E. Haslam, Liming Liang, Katherine L. Tucker, Kaumudi Joshipura, Shilpa N. Bhupathiraju

**Affiliations:** 1 Channing Division of Network Medicine, Brigham and Women’s Hospital and Harvard Medical School, 181 Longwood Ave, Boston, MA 02115, USA; nhcsa@channing.harvard.edu (C.M.S.); nhdah@channing.harvard.edu (D.E.H.); 2 Department of Epidemiology, Harvard T.H. Chan School of Public Health, Boston, MA 02115, USA; zheyicao@hsph.harvard.edu; 3 Department of Nutrition, Harvard T.H. Chan School of Public Health, Boston, MA 02115, USA; lpacheco@hsph.harvard.edu; 4 Department of Surgical Sciences, School of Dental Medicine, University of Puerto Rico, San Juan, PR 00921, USA; sona.tumanyan@upr.edu (S.R.-T.); kaumudi.joshipura@upr.edu (K.J.); 5 Department of Biostatistics, Harvard T.H. Chan School of Public Health, Boston, MA 02115, USA; lliang@hsph.harvard.edu; 6 Department of Biomedical and Nutritional Sciences and Center for Population Health, University of Massachusetts, Lowell, MA 01854, USA; katherine_tucker@uml.edu

**Keywords:** cardiometabolic risk factors, gut microbiota metabolites, type 2 diabetes, betaine, glycemia, Puerto Rican

## Abstract

(1) Aims: Gut microbiota metabolites may play integral roles in human metabolism and disease progression. However, evidence for associations between metabolites and cardiometabolic risk factors is sparse, especially in high-risk Hispanic populations. We aimed to evaluate the cross-sectional and longitudinal relationships between gut microbiota related metabolites and measures of glycemia, dyslipidemia, adiposity, and incident type 2 diabetes in two Hispanic observational cohorts. (2) Methods: We included data from 670 participants of the Boston Puerto Rican Health Study (BPRHS) and 999 participants of the San Juan Overweight Adult Longitudinal Study (SOALS). Questionnaires and clinical examinations were conducted over 3 years of follow-up for SOALS and 6 years of follow-up for BPRHS. Plasma metabolites, including L-carnitine, betaine, choline, and trimethylamine *N*-oxide (TMAO), were measured at baseline in both studies. We used multivariable linear models to evaluate the associations between metabolites and cardiometabolic risk factors and multivariable logistic and Poisson regressions to assess associations with prevalent and incident type 2 diabetes, adjusted for potential confounding factors. Cohort-specific analyses were combined using a fixed-effects meta-analysis. (3) Results: Higher plasma betaine was prospectively associated with lower fasting glucose [−0.97 mg/dL (95% CI: −1.59, −0.34), *p* = 0.002], lower HbA1c [−0.02% (95% CI: −0.04, −0.01), *p* = 0.01], lower HOMA-IR [−0.14 (95% CI: −0.23, −0.05), *p* = 0.003], and lower fasting insulin [−0.27 mcU/mL (95% CI: −0.51, −0.03), *p* = 0.02]. Betaine was also associated with a 22% lower incidence of type 2 diabetes (IRR: 0.78, 95% CI: 0.65, 0.95). L-carnitine was associated with lower fasting glucose [−0.68 mg/dL (95% CI: −1.29, −0.07), *p* = 0.03] and lower HbA1c at follow-up [−0.03% (95% CI: −0.05, −0.01), *p* < 0.001], while TMAO was associated with higher fasting glucose [0.83 mg/dL (95% CI: 0.22, 1.44), *p* = 0.01] and higher triglycerides [3.52 mg/dL (95% CI: 1.83, 5.20), *p* < 0.0001]. Neither choline nor TMAO were associated with incident type 2 diabetes. (4) Conclusions: Higher plasma betaine showed consistent associations with a lower risk of glycemia, insulinemia, and type 2 diabetes. However, TMAO, a metabolite of betaine, was associated with higher glucose and lipid concentrations. These observations demonstrate the importance of gut microbiota metabolites for human cardiometabolic health.

## 1. Introduction

The human gut microbiota is a large and intricate symbiotic ecosystem, with growing evidence that its composition and function are associated with host health and chronic disease risk [1]. Studies have shown distinctions in microbial composition or diversity between patients with and without type 2 diabetes and other metabolic diseases [2,3,4]. As metabolic diseases, including cardiovascular disease and type 2 diabetes, are among the leading causes of mortality and morbidity globally [5,6], it is important to understand what role the gut microbiota may play in the progression of such diseases. 

The gut microbiota is an integral part of human metabolism and can modulate host responses to diet [7,8]. Food components that reach the colon are metabolized by microbes, and the resulting metabolite byproducts may be absorbed through the colonic wall, producing a variety of downstream physiological effects. While some microbial metabolites, such as short-chain fatty acids [9,10], have been associated with beneficial effects, others have been indicated as potentially harmful [11]. Gut microbiota are known to metabolize dietary quaternary amines [12], including phosphatidyl choline and its metabolites (choline and betaine, found in eggs, dairy, red meat, poultry, and shellfish) and L-carnitine [13] (found in red meat), into trimethylamine (TMA). TMA is then further oxidized by hepatic enzymes to trimethylamine-*N*-oxide (TMAO), a known pro-atherosclerotic metabolite [13,14]. Plasma TMAO, L-carnitine, betaine, and choline have all been associated with CVD and CVD-related events [12,13] but few have examined prospective changes in cardiometabolic risk factors. 

Evaluating the relationship between gut metabolites and cardiometabolic risk factors is important to further our understanding of disease progression. This is especially important in higher risk and traditionally understudied populations, such as Hispanic populations. Hispanic individuals living in the U.S. have a high prevalence of cardiometabolic risk factors and incidence of diabetes, compared to non-Hispanic White individuals [15]. In particular, Puerto Ricans are the second largest Hispanic sub-group in the US [16], and among Hispanic sub-groups, they have the highest prevalence of type 2 diabetes [17] and higher prevalence of cardiometabolic diseases, along with established health disparities [18,19]. Therefore, the aim of this study was to examine whether baseline circulating L-carnitine, betaine, choline, and TMAO are associated with baseline and longitudinal changes in cardiometabolic risk factors (anthropometrics, glycemia measures, and dyslipidemia measures) as well as prevalent and incident type 2 diabetes in two Puerto Rican cohorts.

## 2. Materials and Methods

### 2.1. Study Participants

We included data from the Boston Puerto Rican Health Study (BPRHS) and the San Juan Overweight Adult Longitudinal Study (SOALS). The BPRHS is a population-based longitudinal cohort comprised of 1500 Puerto Rican adults living in the Greater Boston area, aged 45–75 years. Specific details of the design and recruitment have been published elsewhere [20]. Study participants were assessed at baseline, 2 years, and 6 years with bilingual interviewers. SOALS is a three-year prospective cohort of adults aged 40–65 years in the San Juan area with overweight and obesity [21]. Participants completed baseline and follow-up examinations that included anthropometric measurements, interviewer-administered questionnaires, and fasting laboratory-derived measurements. Study protocols were reviewed and approved by the Institutional Review Boards of Tufts Medical Center and Northeastern University (BPRHS) and of the University of Puerto Rico (SOALS), and all participants of BPRHS and SOALS provided written informed consent. 

### 2.2. Assessment of Plasma Metabolites

Blood samples were collected after 10 h of fasting from participants in both cohorts at baseline. In BPRHS, blood samples were drawn in participants’ homes during the baseline home visit/examination by a certified phlebotomist, separated with a portable centrifuge, transported on dry ice to the laboratory, and stored at −70 °C. In SOALS, blood samples were drawn at the clinical research center by nurses during the baseline clinic visit, processed, and stored at −80 °C. Plasma samples were blindly assayed by Metabolon, Inc. (Durham, NC, USA) using ultrahigh-performance liquid chromatography-tandem mass spectroscopy (UPLC-MS) [22]. Chemical peaks were identified using Metabolon’s software and library of known metabolites and recurrent unknown entities, based on authenticated standards and information on retention time/index, mass to charge ratio, and chromatographic data. Peaks are quantified using area-under-the-curve. Internal quality control samples were injected in random order along with samples. Undetectable values were imputed at a value equal to half the minimum of each measured metabolite. All metabolite levels were transformed using inverse normal transformation. In this analysis, we focus on 4 gut microbiota-related metabolites: L-carnitine, betaine, choline, and trimethylamine *N*-oxide (TMAO). We also examine the ratio of betaine to choline, as choline is the direct precursor of betaine, and these metabolites have previously been associated with cardiometabolic risk in opposite directions [23,24].

### 2.3. Assessment of Type 2 Diabetes

In the BPRHS, type 2 diabetes was defined at baseline, 3-year follow-up, and 6-year follow-up as having fasting glucose ≥ 126 mg/dL, HbA1c ≥ 6.5%, or use of blood-sugar lowering medication. In SOALS, type 2 diabetes was defined at baseline as having fasting glucose ≥ 126 mg/dL, 2 h post-load glucose ≥ 200 mg/dL, or HbA1c ≥ 6.5%. At the 3-year follow-up, type 2 diabetes was defined by the same criteria or as a report of a diagnosis by a physician. 

### 2.4. Assessment of Cardiometabolic Markers

Cardiometabolic markers of interest were measured at baseline and follow-up exams and included measures of glycemia (glucose, hemoglobin A1c, HOMA-IR, and insulin), dyslipidemia (low-density lipoprotein [LDL-C], high-density lipoprotein cholesterol [HDL-C], and triglyceride concentrations), *C*-reactive protein (CRP), and anthropometrics (BMI, waist circumference, and weight).

Plasma glucose was measured using Vitros System 150 (intra-assay coefficient of variation: 1.21%; inter-assay CV: 3.06%) in SOALS and using Olympus AU400e (Olympus America Inc., Melville, NY, USA; intra-assay CV: 2%; inter-assay CV: 3.4%) in BPRHS. Plasma insulin was measured using a TOSOH analyzer immune-enzymometric assay in SOALS and using the Insulin Kit (LKIN1) on the Immulite 1000 (Seimens Medical Solutions Diagnostics, Los Angeles, CA, USA, in BPRHS. HOMA-IR was calculated with the equation: [fasting glucose (mg/dL) × fasting insulin (mg/dL)]/405 [25]. HbA1c was measured by the latex immunoagglutination inhibition method on the DCA 2000+ Analyzer (Siemens Healthcare Diagnostics, Tarrytown, NY, USA) in SOALS and by the latex-enhanced immunoturbimetric method on the Cobas FARA using the Roche Unimate HbA1c kit (Roche Diagnostics, Indianapolis, Indiana) in BPRHS. 

HDL-C and triglycerides were assessed using enzymatic assays (Roche Diagnostics, Indianapolis, IN, USA) in SOALS and using the Olympus AU400e in BPRHS. LDL-C was calculated using the Friedewald equation in both studies [26]. High-sensitivity CRP was measured using the latex turbidimetric method by Beckman Coulter AU5421 K-assay (Beckman Coulter, Inc., Brea, CA, USA) in SOALS and by the Immulite 1000 High Sensitivity CRP Kit (LKCRP1) on the Immulite 1000 in BPRHS.

### 2.5. Covariate Assessment

Potential confounders, such as age, sex, smoking status, education, income, family history of diabetes (SOALS only), alcohol consumption, multivitamin use (BPRHS only), perceived stress score (BPRHS only), language acculturation score (BPRHS only), and medication use (hypertension, glucose-lowering, and lipid-lowering), were collected by interviewer-administered questionnaires at baseline in both cohorts. Diet quality was assessed using the American Heart Association (AHA) diet score [27] using data from validated food frequency questionnaires in BPRHS [28]. Height, weight, and waist circumference were measured 2–3 times using standard methods, and measures were averaged. BMI was calculated as: [weight (kg)]/[height (cm)]^2^. Physical activity was assessed using a modified version of the Paffenbarger questionnaire in BPRHS. In SOALS, a metabolic equivalent of task score (MET) was calculated based on self-reported time and frequency of physical activities during a typical week.

### 2.6. Statistical Analysis

A total of 736 participants from BPRHS and 1191 participants from SOALS had measures of circulating metabolites at baseline and completed follow-up exams (Figure S1). Participants were further excluded if they had missing information on type 2 diabetes or confounders (*n* = 66 in BPRHS; *n* = 192 in SOALS). This left a sample size of *n* = 670 in BPRHS and *n* = 999 for baseline cross-sectional analyses. For analyses on incident type 2 diabetes, we additionally excluded participants who met the criteria for type 2 diabetes at baseline, resulting in sample sizes of *n* = 316 with 43 incident cases of type 2 diabetes in BPRHS and *n* = 924 with 63 incident cases of type 2 diabetes in SOALS. We used multivariable linear regression models to evaluate the cross-sectional and longitudinal associations between baseline metabolites and cardiometabolic risk factors. Models included adjustments for age, sex, diabetes status, smoking status, education, physical activity score, alcohol intake, hypertension medication use and lipid-lowering medication use, BMI, and waist circumference (or change in waist circumference for longitudinal analyses). In BPRHS, models were additionally adjusted for income, acculturation, perceived stress score, multivitamin use, and the American Heart Association diet score [27]. Longitudinal models of the follow-up measures were additionally adjusted for the baseline value of the outcome variable. Associations with type 2 diabetes were assessed using logistic regression at baseline and Poisson regression at follow-up and adjusted for the same covariates (except for diabetes status, as those with diabetes at baseline were excluded). Influential outliers were identified through the analysis of residuals and were excluded from the final analysis if the Cook’s Distance value exceeded 0.5. Cohort-specific estimates were combined using inverse-variance-weighted fixed-effects meta-analyses. For outcomes where significant heterogeneity between studies was detected, a random-effects meta-analysis was used. Regression analyses were conducted in SAS statistical software version 9.4 (SAS Institute, Cary, NC, USA), and meta-analysis was conducted using R (V.4.1.3, R Core Team, Vienna, Austria), package meta, developed by Guido Schwarzer [29].

## 3. Results

### 3.1. Baseline Characteristics

A total of 670 participants were included from BPRHS and 999 participants were included from SOALS. Cohorts had similar baseline mean age, BMI, and sex distribution (Table 1). Participants in SOALS tended to have higher education, were less likely to be current smokers, and were less active compared to the participants in BPRHS.

### 3.2. Cardiometabolic Risk Factors

In multivariable adjusted cross-sectional meta-analyses (Table S1), each 1 SD higher L-carnitine was associated with 0.48 mg/dL higher fasting insulin (95% CI: 0.10, 0.86), −0.67 mg/dL lower HDL cholesterol (95% CI: −1.26, −0.07), 2.36 mg/dL higher LDL cholesterol (95% CI: 0.74, 3.99), and −0.48 mg/L lower CRP (95% CI: −0.79, −0.17). Higher plasma choline was associated with 8.14 mg/dL higher triglycerides (95% CI: 3.28, 13.0), 0.44 mg/L higher CRP (95% CI: 0.10, 0.78), 2.42 cm higher waist circumference (95% CI: 1.72, 3.13), and 1.20 kg/m^2^ higher BMI (95% CI: 0.89, 1.52). Higher plasma TMAO was also associated with higher CRP [0.35 mg/dL (95% CI: 0.04, 0.66)] and higher waist circumference [1.01 cm (95% CI: 0.33, 1.69)]. On the other hand, higher plasma betaine was associated with favorable cardiometabolic measures: −0.23 lower HOMA-IR (95% CI: −0.34, −0.13), −0.91 mcU/mL lower fasting insulin (95% CI: −1.31, −0.52), 0.92 mg/dL higher HDL cholesterol (95% CI: 0.31, 1.52), and −17.5 mg/dL lower triglycerides (95% CI: −22.1, −12.9). The ratio of betaine to choline was not associated with any cardiometabolic risk factors in cross-sectional analyses.

In the longitudinal multivariable adjusted meta-analyses, higher plasma L-carnitine was associated with −0.68 mg/dL lower fasting glucose (95% CI: −1.29, −0.07) and −0.03% lower HbA1c (95% CI: −0.05, −0.01) at follow-up (Table 2). Similarly, higher plasma betaine was associated with −0.97 mg/dL lower fasting glucose (95% CI: −1.59, −0.34), −0.02% lower HbA1c (95% CI: −0.04, −0.01), −0.14 lower HOMA-IR (95% CI: −0.23, −0.05), and −0.27 mcU/mL lower fasting insulin (95% CI: −0.51, −0.03) at follow-up. Neither L-carnitine nor betaine were associated with changes in measures of dyslipidemia, inflammation, or anthropometrics. On the other hand, higher plasma TMAO was associated with higher fasting glucose [0.83 mg/dL (95% CI: 0.22, 1.44)] and higher triglycerides at follow-up [3.52 mg/dL (95% CI: 1.83, 5.20)]. We did not observe significant associations between choline or betaine–choline ratio with any of the cardiometabolic measures, and we did not observe significant associations between any of the metabolites and changes in waist circumference or body weight in longitudinal meta-analyses.

### 3.3. Prevalent and Incident Type 2 Diabetes

**Figure 1:**
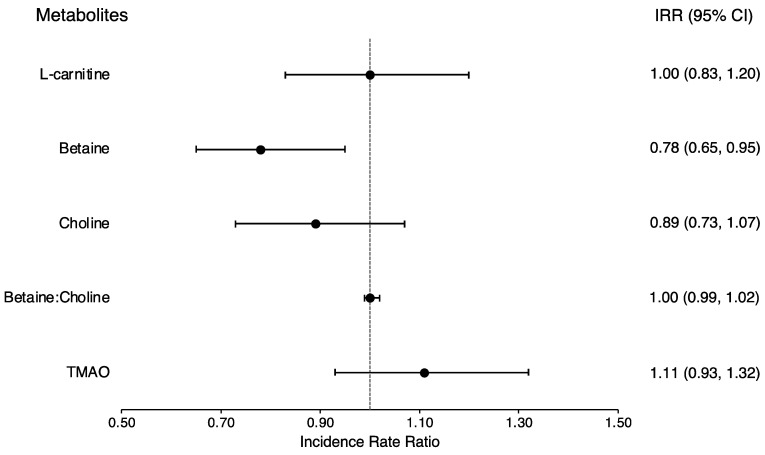
Meta-analysis of incident type 2 diabetes associated with gut microbiota metabolites in the Boston Puerto Rican Health Study (BPRHS) and the San Juan Overweight Adult Longitudinal Study (SOALS). Models adjusted for age, sex, smoking status, education, physical activity score, alcohol intake, income, acculturation (BPRHS only), perceived-stress score (BPRHS only), multivitamin use (BPRHS only), American Heart Association diet score (BPRHS only), hypertension medication use, lipid-lowering medication use, body mass index, and waist circumference change from baseline. BPRHS, Boston Puerto Rico Health Study; SOALS, San Juan Overweight Adults Longitudinal Study; Betaine: choline, betaine–choline ratio; TMAO, trimethylamine *N*-oxide.

In baseline multivariable meta-analyses, each SD higher plasma betaine was associated with a 21% lower likelihood of prevalent type 2 diabetes (IRR: 0.79; 95% CI: 0.69, 0.92) (Table S2). On the other hand, higher plasma TMAO was associated with a 21% higher likelihood of prevalent type 2 diabetes (IRR: 1.21; 95% CI: 1.05, 1.40). Prospective analyses showed similar significant results for plasma betaine but not for TMAO. Higher plasma betaine was associated with a 22% lower incidence of type 2 diabetes (IRR: 0.78; 95% CI: 0.65, 0.95) (Figure 1). There were no significant associations observed for L-carnitine, choline, or the ratio of betaine to choline, either cross-sectionally at baseline or prospectively.

## 4. Discussion

In this meta-analysis of two Puerto Rican cohorts totaling 1669 participants, we observed several significant associations between gut microbiome metabolites and cardiometabolic risk factors. Plasma betaine was significantly associated with lower measures of glycemia and insulinemia at follow-up and with a lower risk of incident type 2 diabetes. Additionally, longitudinal associations included plasma L-carnitine with lower measures of glycemia and TMAO with higher fasting glucose and triglyceride concentrations at follow-up. 

The association between TMAO and increased cardiometabolic risk has been demonstrated in many studies [12,13,14,30,31,32], which aligns with our observations between higher TMAO and higher fasting triglyceride concentration at follow-up. However, while much of the existing literature indicates that TMAO and its precursors (L-carnitine, choline, and betaine) are associated with increased cardiometabolic risk, not all studies have observed clear associations [33,34,35]. One prospective study by Wang et al. found that the association between circulating betaine and choline and the risk of major adverse cardiac events was dependent on concurrent elevated plasma TMAO [30]. Other studies have indicated that plasma betaine may have differential associations in type 2 diabetes, where higher betaine is associated with greater cardiometabolic risk among those with type 2 diabetes, but lower betaine is associated with higher cardiometabolic risk in those without type 2 diabetes [31,36]. Plasma betaine and choline have been prospectively associated with greater insulin sensitivity [37], and dietary choline and betaine intake have also been associated with lower fasting glucose, insulin, and HOMA-IR in a Canadian population [38]. Additionally, L-carnitine has been associated with improved glycemia and lipidemia measures [39], and supplementation with L-carnitine has been suggested as a potential tool for the management of type 2 diabetes [40,41]. This is further supported by our observations of the association between higher plasma L-carnitine and beneficial changes in fasting glucose and HbA1c. 

The relationship between TMAO and type 2 diabetes is also unclear, with studies finding inconsistent results. Evidence from cross-sectional studies has shown higher plasma TMAO in those with type 2 diabetes compared to those without [42,43,44] and higher serum TMAO was associated with a higher risk of type 2 diabetes in a prospective study of 2088 Chinese adults [45]. In contrast, one case–cohort analysis using data from 892 participants in the Prevención con Dieta Mediterránea (PREDIMED) trial found that higher plasma TMAO, betaine, and L-carnitine were associated with a lower risk of type 2 diabetes [46]. Other studies have failed to find evidence of an association between TMAO and type 2 diabetes [37,47]. One recent prospective study of 4442 US adults in the Cardiovascular Health Study (CHS) found no association between TMAO, carnitine, choline, or betaine with incident type 2 diabetes [37]. This aligns with the present study, which did not find evidence for an association between TMAO, L-carnitine, or choline and incident type 2 diabetes. However, we did observe that higher TMAO was associated with increased fasting glucose at follow-up. To our knowledge, only one other prospective study has examined the relationship between plasma TMAO and fasting plasma glucose [48]. Among 300 men and women free from diabetes in the Oral Infections Glucose Intolerance and Insulin Resistance Study (ORIGINS), TMAO was nonlinearly associated with the prevalence of pre-diabetes at baseline but was not associated with plasma glucose at 2-year follow-up. The variability in the literature around TMAO and diabetes may be partly due to differences in study design and study populations. Our study was larger, and although participants in ORIGINS were reportedly nearly 50% Hispanic, they were notably younger (mean age: 34.1 ± 9.9 years) than our participants. It has also been reported that TMAO concentrations can vary greatly in people who are overweight and have diabetes [49], and further research is needed to understand these variations. 

In contrast, we consistently observed that higher plasma betaine was associated with improved measures of glycemia and a lower likelihood of type 2 diabetes, both cross-sectionally and longitudinally. These observations align with the results from the previously mentioned PREDIMED study and among Dutch men and women from the Prevention of Renal and Vascular End-Stage Disease (PREVEND) prospective cohort [50]. Additionally, a recent meta-analysis of metabolomics and type 2 diabetes also found that higher circulating betaine was associated with lower type 2 diabetes risk (RR per 1 SD: 0.82, 95% CI: 0.76–0.89, *I*^2^ = 49%, *n* = 10 studies) [46,50,51]. Plasma betaine has been inversely associated with fasting glucose and HbA1c in coronary artery disease patients [52] and with greater insulin sensitivity in the CHS [37].

Betaine is a methyl donor involved in transmethylation pathways (along with choline and folate), where it methylates homocysteine to methionine and also acts as an intracellular osmolyte [53]. Betaine can be derived directly from the diet, from foods such as spinach, whole grains, eggs, and shellfish, or through the oxidation of choline. Gut microbiota may also contribute to the production of betaine, as evidenced by studies of bran-fed mice [54]. Studies in betaine-supplemented mice have demonstrated that betaine may improve glucose homeostasis via upregulation of fibroblast growth factor 21 (Fgf21) [55], an important metabolic regulator, reduce intramyocellular lipid accumulation and insulin resistance [56], and alter the composition of the gut microbiota to favor species associated with obesity prevention [54]. 

This study benefited from prospective data from two large Puerto Rican cohorts. The cohorts contained detailed information on cardiometabolic risk measures, metabolomic data, and important confounders. However, although we adjusted for potential confounders, we cannot rule out the possibility of residual confounding. Measurement errors, especially in self-reported data, are possible but would likely attenuate observations. Further, the analyses on incident type 2 diabetes may be limited by the shorter available follow-up time, especially in SOALS, which only has 3 years of follow-up. Lastly, generalizability is limited to Puerto Rican populations, and future research would benefit from exploring these relationships in other Hispanic and non-Hispanic populations. 

## 5. Conclusions

In conclusion, we found that gut microbiota metabolites may have differential associations with cardiometabolic risk in two populations of Puerto Ricans. While TMAO may be associated with higher glucose and lipids, betaine, a TMAO precursor, may contribute to a lower risk of glycemia, insulinemia, and type 2 diabetes. Although more research is needed to better understand the specific mechanisms that lead to these differential associations, this study supports growing evidence that circulating TMAO and betaine may be potential biomarkers of interest in the development of type 2 diabetes.

## Figures and Tables

**Table 1:** Baseline characteristics of participants in the Boston Puerto Rican Health Study (BPRHS) and the San Juan Overweight Adult Longitudinal Study (SOALS).

	BPRHS (*n* = 670)	SOALS (*n* = 999)
	Mean (SD) or *n* (%)	Mean (SD) or *n* (%)
Age, years	57.2 (7.41)	50.7 (6.77)
Female, *n* (%)	502 (74.9)	729 (73.0)
Total income, USD	18,003 (18,221)	-
<20,000, *n* (%)	-	543 (54.4)
20,000–49,999, *n* (%)	-	338 (33.8)
≥50,000, *n* (%)	-	118 (11.8)
Education, *n* (%)		
No schooling—7th to 8th grade	336 (50.2)	113 (11.3)
9th–12th grade	237 (35.4)	439 (43.9)
Some college or more	97 (14.5)	447 (44.7)
Smoking status, *n* (%)		
Never	315 (47.0)	639 (64.0)
Past	204 (30.5)	179 (17.9)
Current	151 (22.5)	181 (18.1)
Alcohol consumption status, *n* (%)		
Never	203 (30.3)	442 (44.2)
Past	197 (29.4)	113 (11.3)
Current	270 (40.3)	444 (44.4)
Multivitamin use, *n* (%)	134 (20.0)	-
Lipid-lowering medication use, *n* (%)	295 (44.0)	85 (8.51)
Hypertension medication use, *n* (%)	376 (56.1)	267 (26.73)
BMI, kg/m^2^	32.2 (6.67)	33.3 (6.17)
Waist circumference, cm	102 (14.9)	106 (13.98)
LDL cholesterol, mg/dL	108 (34.4)	123 (32.7)
HDL cholesterol, mg/dL	45.2 (12.4)	48.1 (13.1)
Triglycerides ^a^, mg/dL	162 (112)	149 (83.7)
Glucose, mg/dL	120 (50.2)	95.8 (20.2)
Hemoglobin A1c, %	7.00 (1.78)	5.80 (0.62)
HOMA-IR	6.06 (9.99)	2.62 (1.83)
Insulin ^a^, mcU/mL	18.8 (26.2)	10.8 (6.83)
*C*-reactive protein, mg/L	6.36 (8.83)	5.92 (6.32)
Systolic blood pressure, mmHg	136 (18.8)	129 (17.1)
Diastolic blood pressure, mmHg	81.5 (10.7)	80.9 (9.67)
Physical activity score	31.4 (4.40)	22.0 (39.7)
Alcohol, g/d	4.05 (15.4)	2.36 (5.82)
AHA diet score	8.70 (2.04)	-
Psychosocial stress score	23.4 (9.67)	-
Language acculturation score	22.6 (21.2)	-

Values are means (SD) unless otherwise noted. ^a^ Geometric mean and interquartile range. AHA, American Heart Association.

**Table 2:** Longitudinal meta-analysis of gut microbiota metabolites and changes in measures of cardiometabolic risk factors in the Boston Puerto Rican Health Study (BPRHS) and the San Juan Overweight Adult Longitudinal Study (SOALS).

	L-Carnitine	Betaine	Choline	Betaine:Choline	TMAO
**Glycemia**					
**HOMA-IR**	0.05 (−0.03; 0.14)	**−0.14** **(−0.23; −0.05)**	−0.01 (−0.11; 0.08)	−0.003 (−0.01; 0.004)	0.04 (−0.05; 0.13)
**Insulin, mcU/mL**	0.14 (−0.10; 0.37)	**−0.27** **(−0.51; −0.03)**	0.01 (−0.24; 0.26)	−0.01 (−0.02; 0.01)	0.13 (−0.10; 0.36)
**Glucose, mg/dL**	**−0.68** **(−1.29; −0.07)**	**−0.97** **(−1.59; −0.34)**	0.46 (−0.20; 1.12)	−0.01 (−0.06; 0.03)	**0.83** **(0.22; 1.44)**
**HbA1c, %**	**−0.03** **(−0.05; −0.01)**	**−0.02** **(−0.04; −0.01)**	0.01 (−0.01; 0.03)	0.001 (−0.001; 0.002)	0.01 (−0.01; 0.03)
**Dyslipidemia and Inflammation**					
**HDL-C, mg/dL**	−0.04 (−0.24; 0.16)	0.08 (−0.13; 0.28)	−0.13 (−0.35; 0.09)	−0.01 (−0.03; 0.002)	−0.16 (−0.36; 0.05)
**LDL-C, mg/dL**	−0.10 (−0.80; 0.60)	0.58 (−0.12; 1.28)	−0.63 (−1.38; 0.13)	−0.02 (−0.07; 0.04)	−0.44 (−1.15; 0.26)
**Triglycerides, mg/dL**	1.09 (−0.59; 2.77)	0.09 (−1.65; 1.82)	1.40 (−0.42; 3.21)	−0.06 (−0.19; 0.07)	3.52 (1.83; 5.20)
**CRP, mg/L**	−0.10 (−0.27; 0.08)	0.04 (−0.14; 0.22)	0.01 (−0.18; 0.20)	0.01 (−0.04; 0.06) *	0.07 (−0.10; 0.25)
**Anthropometrics**					
**Waist, cm**	0.13 (−0.14; 0.41)	−0.02 (−0.29; 0.26)	0.07 (−0.23; 0.36)	−0.01 (−0.03; 0.01)	0.12 (−0.15; 0.39)
**Weight, kg**	0.09 (−0.13; 0.31)	0.17 (−0.05; 0.39)	−0.04 (−0.27; 0.20)	−0.07 (−0.19; 0.05) *	−0.20 (−0.42; 0.03)

Values represent the unit change in outcome per 1 SD increase in metabolite. Models adjusted in both studies for age, sex, diabetes status, smoking status, education, income, physical activity score, alcohol intake, anti-hypertension medication use, lipid-lowering medication use, body mass index (except for waist and weight outcomes), change in waist circumference (except for waist and weight outcomes), and baseline biomarker values; BPRHS only additionally adjusted for multivitamin use, acculturation, perceived stress score, and American Heart Association diet score. *p*-value < 0.05 is bolded; * random-effects meta-analysis result reported due to significant heterogeneity. BPRHS, Boston Puerto Rico Health Study; SOALS, San Juan Overweight Adults Longitudinal Study; Betaine: Choline, betaine-choline ratio; TMAO, trimethylamine *N*-oxide; HDL-C, high-density lipoprotein cholesterol; LDL-C, low-density lipoprotein cholesterol; CRP, c-reactive protein.

## Data Availability

Availability of data and materials: Data are available upon reasonable request, following approval of the analysis plan from the study investigators. For more information on requesting data from the BPRHS (https://www.uml.edu/Research/UML-CPH/Research/bprhs, accessed on 23 March 2024) and SOALS (http://soals.rcm.upr.edu, accessed on 23 March 2024) can be found please refer to on their respective websites.

## Supplementary Materials

The following supporting information can be downloaded at: https://www.mdpi.com/article/10.3390/nu16070959/s1, Figure S1: Exclusions for cross-sectional and longitudinal analyses; Table S1: Cross-sectional meta-analysis of gut microbiota metabolites and measures of cardiometabolic risk factors in BPRHS and SOALS; Table S2: Meta analysis of cross-sectional association between gut microbiota metabolites and likelihood of prevalent type 2 diabetes [IRR (95% CI)] in the BPRHS and SOALS; Table S3: Cohort-specific cross-sectional linear associations between gut microbiota metabolites and measures of cardiometabolic risk factors in the BPRHS and SOALS; Table S4: Cohort-specific longitudinal linear association between gut microbiota metabolites and measures of cardiometabolic risk factors in the BPRHS and SOALS.

## Abbreviations

AHA, American Heart Association; BPRHS, Boston Puerto Rican Health Study; CRP, c-reactive protein; Fgf21, fibroblast growth factor 21; HDL-C, high-density lipoprotein cholesterol; LDL-C, low-density lipoprotein cholesterol; MET, metabolic equivalent score; SOALS, San Juan Overweight Adult Longitudinal Study; TMA, trimethylamine; TMAO, trimethylamine *N*-oxide
