# Supplementary material for: Association of Gut Microbiota-Related Metabolites and Type 2 Diabetes in Two Puerto Rican Cohorts
Source: Nutrients. 2024 Mar 27;16(7):959. doi: 10.3390/nu16070959 (PMC11013596; doi:10.3390/nu16070959)
Supplement: Supplementary file 1 [file nutrients-16-00959-s001.zip › nutrients-2914657-supplementary.pdf]

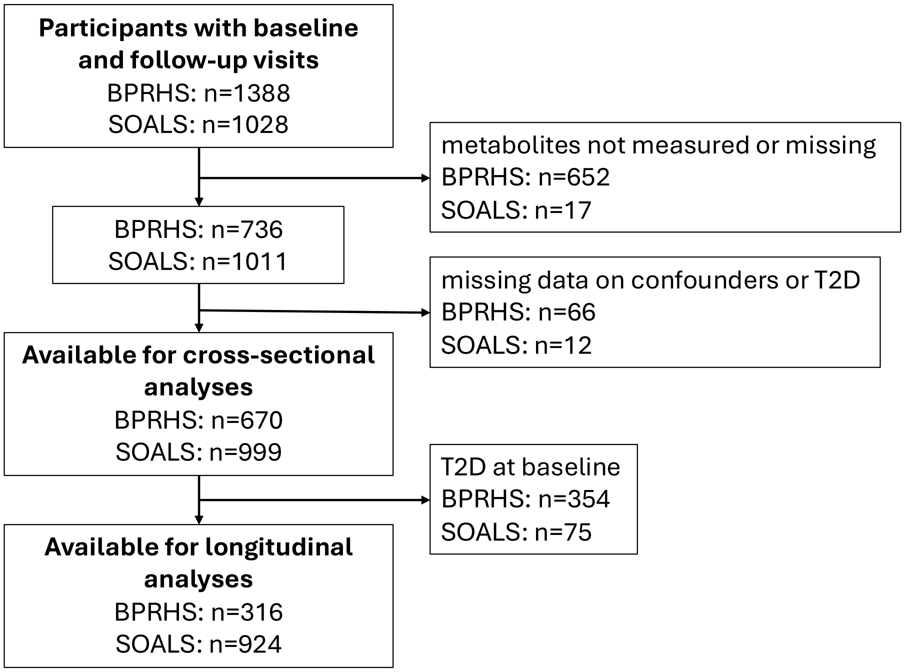

Figure S1. Exclusions for cross-sectional and longitudinal analyses

Table S1. Cross-sectional meta-analysis of gut microbiota metabolites and measures of cardiometabolic risk factors in BPRHS and SOALS

	**L-carnitine**	**Betaine**	**Choline**	**Betaine:Choline**	**TMAO**
**GLYCEMIA**					
**Homa-IR**	0.07 (-0.03; 0.17)	**-0.23 (-0.34; -0.13)**	-0.07 (-0.18; 0.03)	-0.01 (-0.01; 0.002)	0.09 (-0.01; 0.18)
**Insulin, mcU/mL**	**0.48 (0.10; 0.86)**	**-0.91 (-1.31; -0.52)**	-0.23 (-0.65; 0.19)	-0.02 (-0.05; 0.01)	0.36 (-0.03; 0.74)
**Glucose, mg/dL**	-4.64 (-12.6; 3.35)*	-0.86 (-1.88; 0.17)	-0.41 (-1.49; 0.67)	-0.02 (-0.10; 0.06)	0.26 (-0.73; 1.26)
**HbA1c, %**	-0.02 (-0.05; 0.01)	-0.02 (-0.05; 0.02)	0.004 (-0.03; 0.04)	-0.001 (-0.003; 0.002)	0.02 (-0.01; 0.05)
**DYSLIPIDEMIA AND INFLAMMATION**					
**HDL-C, mg/dL**	**-0.67 (-1.26; -0.07)**	**0.92 (0.31; 1.52)**	0.57 (-0.07; 1.20)	0.03 (-0.01; 0.08)	-0.54 (-1.14; 0.06)
**LDL-C, mg/dL**	**2.36 (0.74; 3.99)**	-0.51 (-2.18; 1.16)	0.58 (-1.17; 2.34)	0.05 (-0.08; 0.18)	-0.70 (-2.34; 0.95)
**Triglycerides, mg/dL**	3.27 (-1.26; 7.80)	**-17.5 (-22.1; -12. 9)**	**8.14 (3.28; 13.0)**	-0.21 (-0.57; 0.15)	4.21 (-0.33; 8.75)
**CRP, mg/L**	**-0.48 (-0.79; -0.17)**	0.08 (-0.24; 0.40)	**0.44 (0.10; 0.78)**	0.003 (-0.02; 0.03)	**0.35 (0.04; 0.66)**
**ANTHROPOMETRICS**					
**Waist, cm**	-0.05 (-0.73; 0.63)	-0.06 (-0.75; 0.64)	**2.42 (1.72; 3.13)**	-0.01 (-0.06; 0.05)	**1.01 (0.33; 1.69)**
**BMI, kg/m^2**	0.07 (-0.23; 0.37)	0.09 (-0.22; 0.40)	**1.20 (0.89; 1.52)**	0.01 (-0.02; 0.03)	0.29 (-0.01; 0.59)

Values represent unit difference in outcome per SD increase in metabolite.
Models adjusted in both studies for age, sex, diabetes status, smoking status, education, income, physical activity score, alcohol intake, anti-hypertension medication use, lipid-lowering medication use, body mass index (except for waist and BMI outcomes), and waist circumference (except for waist and BMI outcomes); BPRHS only additionally adjusted for multivitamin use, acculturation, perceived-stress score, and American Heart Association diet score.
P-value <0.05 is bolded; *random-effects meta-analysis result reported due to significant heterogeneity.
BPRHS, Boston Puerto Rico Health Study; SOALS, San Juan Overweight Adults Longitudinal Study; Betaine: Choline, betaine - choline ratio; TMAO, trimethylamine N-oxide; HDL-C, high-density lipoprotein cholesterol; LDL-C, low-density lipoprotein cholesterol; CRP, c-reactive protein.

Table S2. Meta analysis of cross-sectional association between gut microbiota metabolites and likelihood of prevalent type 2 diabetes [IRR (95% CI)] in the BPRHS and SOALS

**Metabolite**	**Tertile 2**	**Tertile 3**	**Per SD**
**L-carnitine**			
BPRHS	0.79 (0.53, 1.18)	**0.61 (0.41, 0.91)**	**0.82 (0.69, 0.97)**
SOALS	0.91 (0.49, 1.68)	1.09 (0.60, 1.97)	0.97 (0.76, 1.25)
Pooled	0.83 (0.59, 1.15)	0.73 (0.53, 1.02)	0.86 (0.75, 0.99)
**Betaine**			
BPRHS	0.69 (0.46, 1.03)	0.71 (0.47, 1.07)	0.85 (0.72, 1.01)
SOALS	**0.52 (0.29, 0.93)**	**0.34 (0.18, 0.65)**	**0.68 (0.52, 0.88)**
Pooled	**0.63 (0.45, 0.88)**	**0.58 (0.41, 0.81)**	**0.79 (0.69, 0.92)**
**Choline**			
BPRHS	1.47 (0.96, 2.24)	1.32 (0.86, 2.02)	**1.23 (1.03, 1.47)**
SOALS	1.01 (0.57, 1.80)	0.59 (0.31, 1.13)	**0.72 (0.55, 0.95)**
Pooled	1.29 (0.92, 1.81)	1.03 (0.72, 1.47)	1.05 (0.91, 1.22)
**Betaine:Choline**			
BPRHS	0.75 (0.50, 1.14)	0.99 (0.66, 1.48)	1.00 (0.99, 1.01)
SOALS	1.27 (0.73, 2.21)	0.68 (0.36; 1.28)	1.00 (0.98, 1.02)
Pooled	0.91 (0.65, 1.26)	0.89 (0.63, 1.25)	1.00 (0.99, 1.01)
**TMAO**[b]			
BPRHS	1.52 (0.99, 2.32)	**1.94 (1.28, 2.96)**	**1.33 (1.11, 1.58)**
SOALS	1.46 (0.80, 2.66)	1.07 (0.57, 2.01)	1.01 (0.79, 1.30)
Pooled	**1.50 (1.06, 2.12)**	**1.62 (1.14, 2.30)**	**1.21 (1.05, 1.40)**

BPRHS multivariable model adjusted for: age, sex, smoking status, education, physical activity score, alcohol intake, income, acculturation, perceived-stress score, multivitamin use, and body mass index. SOALS multivariable model adjusted for: age, sex, smoking status, education, physical activity score, alcohol intake, income, and body mass index.
BPRHS, Boston Puerto Rico Health Study; SOALS, San Juan Overweight Adults Longitudinal Study; Betaine: Choline, betaine - choline ratio; TMAO, trimethylamine N-oxide.
P-value <0.05 is bolded.

Table S3. Cohort-specific cross-sectional linear associations between gut microbiota metabolites and measures of cardiometabolic risk factors in the BPRHS and SOALS

	**L-carnitine**	**Betaine**	**Choline**	**Betaine:Choline**	**TMAO**
**GLYCEMIA**					
**Homa-IR**					
BPRHS	-0.23 (0.38)	-0.16 (0.39)	-0.16 (0.41)	-0.02 (0.03)	**0.82 (0.39)**
SOALS	0.07 (0.05)	**-0.23 (0.05)**	-0.07 (0.06)	-0.01 (0.004)	0.07 (0.05)
**Insulin**					
BPRHS	1.84 (1.03)	-1.81 (1.05)	-0.09 (1.09)	-0.05 (0.08)	**2.32 (1.05)**
SOALS	**0.43 (0.20)**	**-0.88 (0.20)**	-0.23 (0.22)	-0.02 (0.02)	0.29 (0.20)
**Glucose**					
BPRHS	**-8.88 (1.75)**	-2.65 (1.81)	1.08 (1.88)	-0.05 (0.14)	2.66 (1.82)
SOALS	-0.73 (0.53)	-0.69 (0.55)	-0.55 (0.58)	-0.02 (0.04)	0.06 (0.53)
**HbA1c**					
BPRHS	**-0.27 (0.05)**	-0.05 (0.06)	**0.13 (0.06)**	0.002 (0.004)	**0.16 (0.06)**
SOALS	0.002 (0.02)	-0.01 (0.02)	-0.01 (0.02)	-0.001 (0.001)	0.01 (0.02)
**DYSLIPIDEMIA AND INFLAMMATION**					
**HDL-C**					
BPRHS	0.05 (0.46)	**1.61 (0.46)**	0.90 (0.49)	-0.01 (0.04)	-0.35 (0.47)
SOALS	**-1.21 (0.40)**	0.36 (0.42)	0.29 (0.44)	**0.06 (0.03)**	-0.68 (0.40)
**LDL-C**					
BPRHS	**2.70 (1.31)**	-1.49 (1.34)	-1.30 (1.40)	0.10 (0.10)	-1.58 (1.35)
SOALS	**2.14 (1.07)**	0.16 (1.11)	1.89 (1.17)	0.02 (0.09)	-0.14 (1.07)
**Triglycerides**					
BPRHS	-3.91 (4.42)	**-23.3 (4.39)**	1.06 (4.66)	-0.07 (0.35)	7.33 (4.50)
SOALS	**5.97 (2.71)**	**-15.2 (2.76)**	**11.0 (2.93)**	-0.27 (0.22)	3.09 (2.70)
**CRP**					
BPRHS	**-0.95 (0.34)**	0.65 (0.35)	0.27 (0.36)	0.003 (0.03)	0.35 (0.35)
SOALS	-0.35 (0.18)	-0.09 (0.19)	**0.49 (0.20)**	0.003 (0.01)	0.35 (0.18)
**ANTHROPOMETRICS**					
**Waist**					
BPRHS	-0.46 (0.56)	0.15 (0.57)	**3.02 (0.56)**	0.002 (0.04)	0.59 (0.57)
SOALS	0.20 (0.44)	-0.19 (0.46)	**2.00 (0.47)**	-0.01 (0.04)	**1.26 (0.44)**
**BMI**					
BPRHS	-0.13 (0.25)	0.15 (0.25)	**1.39 (0.25)**	0.004 (0.02)	0.14 (0.25)
SOALS	0.20 (0.20)	0.05 (0.20)	**1.07 (0.21)**	0.01 (0.02)	**0.39 (0.20)**

Values represent unit difference in outcome per SD increase in metabolite.
Models adjusted in both studies for age, sex, diabetes status, smoking status, education, income, physical activity score, alcohol intake, anti-hypertension medication use, lipid-lowering medication use, body mass index (except for waist and weight outcomes), and waist circumference (except for waist and weight outcomes); BPRHS only additionally adjusted for multivitamin use, acculturation, perceived-stress score, and American Heart Association diet score.
P-value <0.05 is bolded.

BPRHS, Boston Puerto Rico Health Study; SOALS, San Juan Overweight Adults Longitudinal Study; Betaine: Choline, betaine - choline ratio; TMAO, trimethylamine N-oxide; HDL-C, high-density lipoprotein cholesterol; LDL-C, low-density lipoprotein cholesterol; CRP, c-reactive protein.

Table S4. Cohort-specific longitudinal linear association between gut microbiota metabolites and measures of cardiometabolic risk factors in the BPRHS and SOALS.

	**L-carnitine**	**Betaine**	**Choline**	**Betaine:Choline**	**TMAO**
**GLYCEMIA**					
**Homa-IR**					
BPRHS	0.27 (0.14)	-0.27 (0.14)	0.20 (0.15)	0.003 (0.01)	0.25 (0.14)
SOALS	0.03 (0.05)	**-0.12 (0.05)**	-0.04 (0.05)	-0.003 (0.004)	0.02 (0.05)
**Insulin**					
BPRHS	0.04 (0.22)	-0.13 (0.21)	0.29 (0.23)	-0.003 (0.02)	**0.43 (0.22)**
SOALS	0.17 (0.14)	**-0.34 (0.15)**	-0.12 (0.15)	-0.01 (0.01)	0.002 (0.14)
**Glucose**					
BPRHS	**-1.28 (0.64)**	-0.77 (0.63)	**2.08 (0.67)**	0.03 (0.05)	**1.25 (0.64)**
SOALS	-0.49 (0.36)	**-1.04 (0.37)**	-0.10 (0.39)	-0.03 (0.03)	0.70 (0.36)
**HbA1c**					
BPRHS	**-0.04 (0.02)**	**-0.03 (0.02)**	0.03 (0.02)	**0.002 (0.001)**	0.02 (0.02)
SOALS	**-0.03 (0.01)**	-0.02 (0.01)	-0.01 (0.01)	-0.0002 (0.001)	0.003 (0.01)
**DYSLIPIDEMIA AND INFLAMMATION**					
**HDL-C**					
BPRHS	-0.09 (0.12)	-0.12 (0.13)	-0.11 (0.13)	**-0.02 (0.01)**	-0.12 (0.13)
SOALS	0.08 (0.19)	**0.54 (0.19)**	-0.19 (0.20)	0.01 (0.02)	-0.23 (0.19)
**LDL-C**					
BPRHS	-0.27 (0.46)	0.40 (0.45)	-0.02 (0.49)	-0.06 (0.03)	-0.58 (0.47)
SOALS	0.17 (0.57)	0.89 (0.58)	**-1.57 (0.62)**	0.06 (0.05)	-0.24 (0.57)
**Triglycerides**					
BPRHS	-0.16 (1.14)	0.31 (1.16)	1.20 (1.22)	-0.02 (0.09)	1.83 (1.16)
SOALS	**2.70 (1.30)**	-0.22 (1.36)	1.66 (1.42)	-0.12 (0.10)	**5.57 (1.28)**
**CRP**					
BPRHS	-0.08 (0.14)	0.17 (0.13)	0.02 (0.14)	**0.03 (0.01)**	-0.09 (0.14)
SOALS	-0.11 (0.12)	-0.07 (0.12)	-0.003 (0.13)	-0.02 (0.01)	0.19 (0.12)
**ANTHROPOMETRICS**					
**Waist**					
BPRHS	0.12 (0.21)	-0.28 (0.21)	-0.16 (0.23)	-0.01 (0.02)	-0.20 (0.22)
SOALS	0.15 (0.18)	0.19 (0.19)	0.24 (0.20)	-0.02 (0.01)	0.34 (0.18)
**Weight**					
BPRHS	0.12 (0.12)	0.17 (0.12)	-0.04 (0.13)	-0.01 (0.01)	**-0.24 (0.12)**
SOALS	-0.11 (0.16)	0.19 (0.17)	-0.13 (0.18)	-0.01 (0.01)	-0.02 (0.16)

Values represent unit change in outcome per SD increase in metabolite.
Models adjusted in both studies for age, sex, diabetes status, smoking status, education, physical activity score, alcohol intake, acculturation, hypertension medication use, lipid-lowering medication use, body mass index (except for anthropometric outcomes), waist circumference (except for anthropometric outcomes), and in BPRHS only for multivitamin use, income, perceived-stress score, and American Heart Association diet score.
P-value <0.05 is bolded.

BPRHS, Boston Puerto Rico Health Study; SOALS, San Juan Overweight Adults Longitudinal Study; Betaine: Choline, betaine - choline ratio; TMAO, trimethylamine N-oxide; HDL-C, high-density lipoprotein cholesterol; LDL-C, low-density lipoprotein cholesterol; CRP, c-reactive protein.
